# Inflammation Is a Mediating Factor in the Association between Lifestyle and Fatigue in Colorectal Cancer Patients

**DOI:** 10.3390/cancers12123701

**Published:** 2020-12-09

**Authors:** Evertine Wesselink, Harm van Baar, Moniek van Zutphen, Meilissa Tibosch, Ewout A. Kouwenhoven, Eric T.P. Keulen, Dieuwertje E. Kok, Henk K. van Halteren, Stephanie O. Breukink, Johannes H. W. de Wilt, Matty P. Weijenberg, Marlou-Floor Kenkhuis, Michiel G. J. Balvers, Renger F. Witkamp, Fränzel J. B. van Duijnhoven, Ellen Kampman, Sandra Beijer, Martijn J. L. Bours, Renate M. Winkels

**Affiliations:** 1 Division of Human Nutrition and Health, Wageningen University, Wageningen, 6708 WE, The Netherlands; harm.vanbaar@wur.nl (H.v.B.); moniek.vanzutphen@wur.nl (M.v.Z.); meilissa.tibosch@wur.nl (M.T.); dieuwertje.kok@wur.nl (D.E.K.); michiel.balvers@wur.nl (M.G.J.B.); renger.witkamp@wur.nl (R.F.W.); franzel.vanduijnhoven@wur.nl (F.J.B.v.D.); ellen.kampman@wur.nl (E.K.); renate.winkels@wur.nl (R.M.W.); 2 Ziekenhuis Groep Twente, Department of Surgery, Almelo, 7600 SZ, The Netherlands; E.Kouwenhoven@zgt.nl; 3 Zuyderland Medical Centre, Department of Gastroenterology, Sittard-Geleen, 6162 BG, The Netherlands; e.keulen@zuyderland.nl; 4 Department of Internal Medicine, Admiraal de Ruyter Ziekenhuis, Goes, 4462 RA, The Netherlands; hk.vanhalteren@adrz.nl; 5 Medical Center, Department of Surgery, Maastricht University, Maastricht, 6229 HX, The Netherlands; s.breukink@mumc.nl; 6 Department of Epidemiology, GROW-School for Oncology and Developmental Biology, Maastricht University, Maastricht, 6200 MD, The Netherlands; mp.weijenberg@maastrichtuniversity.nl (M.P.W.); m.kenkhuis@maastrichtuniversity.nl (M.-F.K.); m.bours@maastrichtuniversity.nl (M.J.L.B.); 7 Medical Centre, Department of Surgery, Radboud University, Nijmegen, 6500 HB, The Netherlands; hans.dewilt@radboudumc.nl; 8 Department of Research & Development, Netherlands Comprehensive Cancer Organisation (IKNL), Utrecht, 3511 DT, The Netherlands; s.beijer@iknl.nl

**Keywords:** fatigue, colorectal cancer, inflammation markers, lifestyle, mediation analyses

## Abstract

**Simple Summary:**

Fatigue is common among colorectal cancer patients. A healthier lifestyle may beneficially affect fatigue, although data are sparse. A healthier lifestyle may result in lower levels of inflammation, which is one of the suggested mechanisms by which lifestyle could influence fatigue. In an observational study, we investigated 1) whether a healthier lifestyle was associated with less fatigue among colorectal cancer patients, and 2) whether this association could be explained by inflammation. We showed that a healthier lifestyle was associated with less fatigue, and that inflammation levels mediated this association. Future intervention studies should investigate whether improving lifestyle after cancer diagnosis results in lowering of inflammation markers and subsequent fatigue.

**Abstract:**

Fatigue is very common among colorectal cancer (CRC) patients. We examined the association between adherence to the World Cancer Research Fund/American Institute for Cancer Research (WCRF/AICR) lifestyle recommendations and fatigue among stage I-III CRC patients, and whether inflammation mediated this association. Data from two prospective cohort studies were used. Adherence to the WCRF/AICR recommendations was expressed as a score ranging from 0–7, and assessed shortly after diagnosis. Six months post-diagnosis, fatigue was assessed with the European Organization for Research and Treatment of Cancer quality of life questionnaire C30 (EORTC QLQ-C30), and in a subpopulation, the plasma levels of inflammation markers (IL6, IL8, TNFα, and hsCRP) were assessed. Multiple linear regression analyses were performed to investigate the association between adherence to the WCRF/AICR recommendations and fatigue. To test mediation by inflammation, the PROCESS analytic tool developed by Hayes was used. A higher WCRF/AICR adherence score was associated with less fatigue six months after diagnosis (*n* = 1417, β −2.22, 95%CI −3.65; −0.78). In the population of analysis for the mediation analyses (*n* = 551), the total association between lifestyle and fatigue was (β −2.17, 95% CI −4.60; 0.25). A statistically significant indirect association via inflammation was observed (β −0.97, 95% CI −1.92; −0.21), explaining 45% of the total association between lifestyle and fatigue (−0.97/−2.17 × 100). Thus, inflammation is probably one of the underlying mechanisms linking lifestyle to fatigue.

## 1. Introduction

Fatigue is one of the most common adverse effects colorectal cancer (CRC) survivors experience, and can be present for years after treatment [1]. During, and shortly after, treatment, the prevalence of fatigue is reported to range from 25% to 99%, depending on cancer stage and/or type of treatment, patient population, and method of assessment [1,2].

Several factors have been identified which may contribute to fatigue among cancer patients, including a more advanced stage of disease, type of treatment (radio- and chemotherapy compared to only surgery), recurrences, more comorbidities, and decreased physical activity [1,3,4,5,6]. A healthier lifestyle has been associated with less fatigue among breast and colorectal cancer survivors [7,8,9], where better lifestyle was operationalized as better adherence to the World Cancer Research Fund/American Institute for Cancer Research (WCRF/AICR) recommendations for cancer prevention, which include guidelines on body weight, physical activity, and a healthy diet [10].

Better adherence to the WCRF/AICR lifestyle recommendations on physical activity, body weight, and healthy diet for cancer prevention may potentially impact fatigue by attenuating systemic inflammatory processes [11,12,13]. First, physical activity, obesity, and diet have been associated with fatigue. Being more physically active, is known to lower fatigue in cancer patients [14]. Being underweight, as well as excessive body weight, might be related to increased fatigue [15,16,17], although this was not observed in all studies [9,18,19]. Limited data support a potential role for nutrition in managing fatigue after cancer diagnosis [20]. Second, physical activity, obesity, and diet have been associated with inflammatory markers such as interleukins (IL). A meta-analysis on physical activity and fatigue among breast cancer survivors [12] showed that exercise decreased levels of IL6, IL8, and tumor necrosis factor alfa (TNFα). Furthermore, exercise might lead to a decrease in C-reactive protein (CRP) levels [21]. A higher BMI has been associated with higher circulating levels of IL6, IL8, TNF-α, and CRP [22,23]. Furthermore, several studies suggested that dietary intake is associated with inflammation [24,25]. Diets high in red, and processed, meat and fast foods are associated with higher levels of systemic inflammation, expressed by amongst others IL6, TNFα, and CRP, while diets high in fruit, vegetables, and unsaturated fatty acids were associated with lower levels of inflammation [24,25]. Third, inflammatory markers such as IL6, IL8, CRP, and TNFα are associated with fatigue in cancer survivors [1,21]. Finally, an anti-inflammatory diet, characterized by a high intake of fruit, vegetables, whole grains, and omega-3 fatty acid-rich foods, reduced fatigue in an experimental study among 30 breast cancer patients, compared to non-diet related advice sessions [26]. Taken together, this could imply a possible role for inflammation as a mediating mechanism in the association between lifestyle and fatigue, although this has, to the best of our knowledge, not been investigated to date.

The aim of the current study was, to examine whether adherence to the WCRF/AICR recommendations for cancer prevention at diagnosis was associated with fatigue experienced six months post-diagnosis among patients with stage I-III CRC. Furthermore, the study aimed to determine whether inflammation (plasma levels of IL6, IL8, TNFα, and hsCRP) at six months post-diagnosis (partly) mediated this association.

## 2. Results

**Figure 1:**
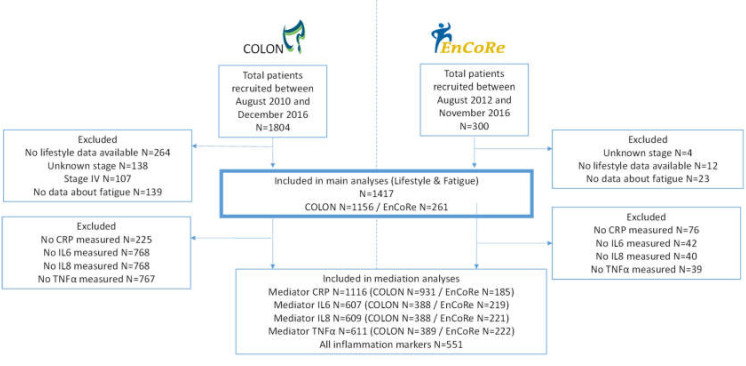
Flow diagram of patients included in this study.

For the analyses regarding the association between lifestyle and fatigue in total, 1417 CRC patients were included; 1156 from the COLON study, and 261 from the EnCoRe study, (Figure 1). Of these patients 514 (36%) were female (Table 1), and median age was 66.1 (IQR 61.2–71.5) years. Two thirds of all patients had colon cancer, 44% were diagnosed with stage III disease, and 30% received chemotherapy, of which 75% received adjuvant chemotherapy. The median WCRF/AICR score at diagnosis was 3.5 (IQR 2.8–4.0), and 26% experienced fatigue at six months post-diagnosis. Patients with the highest adherence to the WCRF/AIRC recommendations, when compared to the lowest adherence, were more often female, were more often highly educated, experienced fatigue less often, and had slightly lower levels of inflammation markers. Baseline characteristics of patients with inflammatory data available (*n* = 607 for cytokines and *n* = 1116 for hsCRP) were similar to the characteristics of patients with no inflammatory data available (Table S2).

In the total population (*n* = 1417), higher adherence to the WCRF/AICR recommendations was statistically significantly associated with less fatigue (β = −2.22, 95% CI: −3.65; −0.78).

For the mediation analyses, we first investigated if a potential mediator was associated with both WCRF/AICR score and fatigue. A healthier lifestyle (higher adherence to the WCRF/AICR recommendations) at diagnosis was associated with lower levels of IL6, TNFα, and hsCRP six months post-diagnosis (β −0.10, 95% CI −0.17; −0.03, β −0.07, 95% CI −0.10; −0.04, β −0.20, 95% CI −0.28; −0.12, respectively). For IL8 the association was not statistically significant (Table 2). IL6 and hsCRP at six months post-diagnosis were statistically significantly associated with higher fatigue levels (β 5.05, 95% CI 2.54; 7.57 and β 3.57, 95% CI 2.37; 4.78, respectively), while IL8 and TNFα were not (Table 3).

In the population of analysis for the mediation analyses (*n* = 551), the total association between lifestyle and fatigue was β −2.17, 95% CI −4.60; 0.25. A statistically significant indirect association (via inflammation) was observed (β −0.97, 95% CI −1.92; −0.21), indicating that a statistically significant part of the association between lifestyle and fatigue was through inflammation. Forty-five percent of the total association between lifestyle and fatigue was mediated by inflammation (−0.97/−2.17 × 100). This mediation was mainly driven by IL6 and hsCRP. The indirect association via IL6 was observed to be β −0.48, 95% CI −1.05; −0.18, indicating a contribution of 22% of IL6 to the total association. The indirect association via hsCRP was observed to be β −0.68 95% CI −1.10; −0.34, 32% of the total association was mediated by hsCRP (Table 4).

Sensitivity analyses, excluding old samples (>2 years stored before analyses, *n* = 47) and outliers (>3SD above the mean, *n* = 10), showed similar results compared to the total population for analyses. 

## 3. Discussion

In the present study we observed a statistically significant association between better adherence to the WCRF/AICR lifestyle recommendations at diagnosis and lower fatigue levels six months post-diagnosis among CRC patients. Furthermore, mediation analyses showed that IL6 and hsCRP were mediators in the association between lifestyle and fatigue.

Our results for the association between adherence to the WCRF/AICR recommendations at diagnosis and lower fatigue levels six months post-diagnosis are in line with the results of two previous observational studies that investigated the association between adherence to the WCRF/AICR recommendations and long-term fatigue in patients with stage I-III CRC [8,9]. Consistently, our results and the results of those studies showed that a favorable lifestyle at, and after, diagnosis was associated with less fatigue in the short and long term in CRC patients.

Our study was the first to assess the mediating effect of inflammation on the association between lifestyle and fatigue. Lifestyle was associated with levels of IL6 and hsCRP, as well as with fatigue. Previous studies showed that nutrition as well as physical activity (both part of the WCRF/AICR score) are associated with inflammation [14,24], and/or fatigue [1,13,21]. Results of our mediation analysis, combining those three factors for the first time (i.e., lifestyle, inflammation, and fatigue), showed that 45% of the association observed between lifestyle and fatigue could be attributed to inflammation (mainly driven by IL6 and hsCRP). In other words, a healthier lifestyle may lead to less fatigue, in part, by lowering inflammation. Of note, we measured the exposure variable (lifestyle at diagnoses) before the outcome (fatigue six months after diagnosis), while the mediator and outcome were measured at the same time-point. Ideally, inflammation should be measured several times between exposure and outcome [28]. Despite this limitation, our results clearly indicate a role for inflammation in the association between lifestyle and fatigue.

The exact biological pathways by which inflammation mediates the association between lifestyle and fatigue in cancer patients are not completely understood. As described before, a healthier lifestyle (e.g., healthy weight, exercise, and a healthy diet) are associated with lower circulating levels of inflammation markers. Inflammation, higher levels of pro-inflammatory cytokines, such as IL6, IL8, hsCRP, or TNFα [1], and an increased activity of NFκB [29], are hypothesized to induce fatigue [1,30,31]. Increasingly, data support the concept that an elevated peripheral inflammatory tone triggers immunological reactions in the central nervous system and changes in metabolic processes, leading to various manifestations of what has been referred to as “sickness behavior” or “sick person syndrome”. Fatigue is considered one of the components of this response. This concept has recently been reviewed by Karshikoff et al. [30] and Lacourt et al. [31]. On a mechanistic basis, we cannot explain why IL6 and hsCRP were observed to be mediators, while IL8 and TNFα were not, since all four are associated with lifestyle, and all four could potentially activate both pathways described above. From a statistical point of view, these non-findings can be explained by the fact that in our study population IL8 and TNFα were not statistically significantly associated with fatigue, and could therefore not be mediators. Despite the inconsistency between the specific markers of inflammation, results of our study showed that systemic low grade inflammation is a mediator in the association between lifestyle and fatigue.

Given the supposed role of inflammation in fatigue, preventing or reversing low grade inflammation is of importance. However, before interventions aimed at reducing inflammation can be conducted, the role of inflammation in fatigue should be further investigated. It should be investigated in an intervention study, whether adopting a healthier lifestyle during and after cancer treatment results in lower levels of inflammation markers (IL6, TNFα), and ultimately less fatigue. If so, personalized lifestyle advice can be given to patients with higher levels of pro-inflammatory cytokines.

This study has several limitations and strengths. First, inflammation data were only available for a subset of our original population, which lowered our statistical power in the mediation analyses. Nevertheless, both IL6 and hsCRP were identified as having a mediating role in the association between adherence to the WCRF/AICR recommendations and fatigue. Furthermore, we used the existing WCRF/AICR recommendation score, in which all recommendations are weighted equally [10]. Although the used WCRF/AICR scoring is simple, and relatively easy to apply, one could debate whether all components of the score contribute equally to fatigue and inflammation. Future studies should assess whether assigning different weighting factors to each recommendation is more appropriate. The strengths of the study are that we had extensive information about lifestyle, clinical variables, circulating levels of inflammation markers, and patient-reported outcomes (such as fatigue), which allowed us to study for the first time the mediating effect of inflammation in the association between lifestyle and fatigue. Our data complement existing evidence regarding lifestyle and fatigue, and the role of inflammation as a mediating factor.

## 4. Materials and Methods

### 4.1. Study Population

For this study, we used data of two prospective cohort studies: the COLON study [32], and the EnCoRe study [33]. For the COLON study, hospital staff of eleven participating hospitals invited eligible patients with stage I–IV of disease, shortly after diagnosis and before scheduled surgery, to participate in the study. Patients were not eligible if they had a history of CRC, a previous (partial) bowel resection, known hereditary CRC, inflammatory bowel disease, dementia, or another mental condition limiting their ability to fill out surveys, or were non-Dutch speaking. For the EnCoRe study, patients with stage I to III CRC from three hospitals were enrolled at diagnosis. Exclusion criteria for the EnCoRe study were diagnosis of stage IV disease; current home address not in the Netherlands; inability to understand the Dutch language in speech, as well as in writing; and presence of comorbidities that might obstruct successful participation, including cognitive disorders such as Alzheimer disease, and severe visibility or hearing disorders, such as complete blindness and/or deafness [33]. In total 1417 CRC patients were included, 1156 from the COLON study, and 261 from the EnCoRe study, see Figure 1. Additional exclusion criteria for the present analyses were: missing data on lifestyle (to build the WCRF/AICR score) (*n* = 272), fatigue six months after diagnosis (*n* = 162), stage of disease (*n* = 138), stage IV CRC (*n* = 107).

The study was performed in accordance with the Declaration of Helsinki. The COLON study (ClinicalTrials.gov Identifier: NCT03191110) was approved by the Committee on Research involving Human Subjects, region Arnhem-Nijmegen, the Netherlands. The EnCoRe study (Netherlands Trialregister number NL6904) was approved by the Medical Ethics Committee of the University Hospital Maastricht and Maastricht University, the Netherlands. All participants of the COLON and EnCoRe studies provided written informed consent.

### 4.2. WCRF/AICR Score

We followed the approach as published previously [10] of scoring adherence to the 2018 WCRF/AICR lifestyle recommendations for cancer prevention [34]. The score includes seven lifestyle recommendations: ”maintain a healthy weight”; “be physically active”; “eat a diet rich in wholegrains, vegetables, fruits, and beans”; “limit consumption of “fast foods”, and other processed foods high in fat, starches or, sugars”; “limit consumption of red and processed meat”; “limit consumption of sugar-sweetened drinks”; and “limit alcohol consumption”. Two recommendations were excluded: the recommendation “for mothers: to breastfeed their baby, if they can” was excluded as it is only specific to mothers who are able to breastfeed; the recommendation about dietary supplement use was not included, since it was not operationalized in the standard WCRF/AICR score [10]. For complete adherence to a recommendation 1 point was assigned, for moderate adherence 0.5 points, and otherwise 0 points. Total scores could range from 0 to 7. In Supplementary Table S1 the exact breakdown of the scoring system is depicted.

Data on body weight, height, and waist circumference were self-reported in the COLON study. In the EnCoRe study, body weight, height, and waist circumference were measured by a research dietician during a home visit. An extended validated semi-quantitative food frequency questionnaire (FFQ) of 204 items for the COLON study [35,36] and 253 items for the EnCoRe study [37] was used to assess habitual dietary intake and consumption of alcoholic drinks in the preceding month (COLON) or year (EnCoRe). Intake of foods and beverages in grams per day was calculated for all food groups (fruit and vegetables, red and processed meat, fast food, alcohol, sugar-sweetened beverages). Energy and nutrient intakes were calculated using the Dutch Food Composition Table 2011 (NEVO table) [38]. Physical activity was assessed using the self-reported short questionnaire to assess health-enhancing physical activity (SQUASH) in both cohorts [39]. Moderate-to-vigorous physical activity in minutes per week was assessed from the data of that questionnaire, in order to score adherence to the physical activity guideline (see Table 1).

### 4.3. Fatigue

Fatigue was assessed using the fatigue domain of the validated European Organization for Research and Treatment of Cancer quality of life questionnaire C30 (EORTC QLQ-C30 version 3.0). Fatigue scores were based on three items in this questionnaire, namely: “Did you need to rest?”; “Have you felt weak?”; and “Were you tired?”. Participants could answer on a four-point Likert scale ranging from “not at all” to “very much”; 1 to 4 points were assigned accordingly. First, a “RawScore” was calculated as the average of the three items. Next, this score was linearly converted to the total fatigue score with a 0–100 scale, as recommended for this questionnaire, where a higher score indicates more fatigue [40]. Within the COLON study, fatigue was assessed six months after diagnosis, within the EnCoRe study, fatigue was assessed six weeks and six months after finalizing treatment. For EnCoRe, therefore, fatigue data closest to six months after diagnosis was selected for the analyses (median 6.8 months, IQR 6.4–8.3). To identify the number of patients with fatigue six months after diagnosis, a fatigue score of >39 was defined as having fatigue [41].

### 4.4. Blood Collection and Inflammatory Markers

Non-fasting blood samples were drawn into EDTA tubes during a regular clinical visit in the hospital (COLON study) or during a home visit (EnCoRe study), around six months after diagnosis. All blood samples were centrifuged, and plasma was aliquoted and stored in a freezer at −80 °C until further analysis.

Due to their possible relation with physical activity [12,21], BMI [22,23], dietary intake [24,25], and fatigue [1,21], the following inflammatory markers were measured: IL6, IL8, TNFα, and high sensitive C-reactive protein (hsCRP).

Levels of IL6, IL8, and TNFα were quantified in plasma using a custom-made multiplex assay, using electrochemiluminescence detection (Meso Scale Diagnostics, Rockville, MD, USA), in our lab at Wageningen University & Research, as described previously [42]. Samples were analyzed in duplicate according to instructions of the manufacturer on a QuickPlex SQ 120 plate reader (Meso Scale Diagnostics). Each sample plate contained a calibration curve and quality control samples provided by the manufacturer, with different levels of each inflammation marker. Target values for the quality control samples were provided. Inter-batch and intra-batch coefficients of variation were <8%, and observed values deviated less than 15% from target values. Analyses of inflammation markers were made in a sub-population (Figure 1), as cytokines were previously shown to remain stable in plasma for a period up to 2 years of storage at −80 °C [43]. Therefore, samples stored >2 years were not analyzed within the COLON study.

Levels of hsCRP were measured using an immuno-MALDI mass spectrometry method [44] (BEVITAL, Bergen, Norway). The inter-assay coefficient of variation ranged between 3 and 6%.

### 4.5. Covariates

Demographic information (including age and sex) and smoking history were collected with self-reported questionnaires. Clinical data, such as stage of disease, tumor location (colon/rectum), type of treatment (surgery, neo-adjuvant/adjuvant chemotherapy, or radiotherapy), and presence of comorbidities (diabetes, endocrine disorders, cardiovascular, infectious, gastro-intestinal, muscular and joint, neurologic, pulmonary and urogenital diseases) were derived from the Dutch ColoRectal Audit (DCRA) [45].

### 4.6. Data Analysis

Population characteristics were described as numbers with percentages, and medians with interquartile range (IQR) for the total population, and stratified by tertiles of adherence to the WCRF/AICR recommendations. Levels of inflammatory markers were log-transformed to obtain normally distributed data.

Descriptive statistics were used to describe demographic, lifestyle, and clinical characteristics of the total population, and patients of whom data about inflammation levels were available, and those of whom not.

Multiple linear regression analyses were used to assess the association of adherence to the WCRF/AICR recommendations at diagnosis (continuous, 1 point increment), and fatigue (continuous, 1 point increment) six months after diagnosis. Betas (β) and 95% confidence intervals (CIs) were reported to describe the associations. All analyses were tested for effect modification by sex, stage of disease, and cohort, by calculating the p value for interaction. None of these variables were identified as effect modifiers. Potential confounders were: age (years), stage of disease (stage I/II/III), sex (male/female), comorbidities (yes/no), smoking (current/former/never), stoma at time of fatigue assessment (yes/no), chemotherapy (yes/no), radiotherapy (yes/no), recurrence within one year after diagnosis, and cohort (COLON/EnCoRe). Variables were included in the final model if they changed the β for fatigue or inflammation by 10% or more when the variable was individually added to a crude model. Age, stage of disease, sex, chemotherapy (yes/no), and cohort (COLON/EnCoRe) were identified as confounders, and included in all models.

### 4.7. Mediation Analyses

Mediation analyses were conducted to analyze whether inflammation six months after diagnosis was a mediator in the association between adherence to the WCRF/AICR recommendations at diagnosis and fatigue six months after diagnosis.

Before performing the mediation analyses, the associations between adherence to the WCRF/AICR recommendations and inflammation markers (exposure–mediator), and the association between inflammation markers and fatigue (mediator–outcome) were assessed using multiple linear regression analyses. The same confounders as identified above for the total associations were included in all models created for the mediation analyses.

Two paths are important in mediation analyses. First, the path from lifestyle to fatigue, without passing through inflammation, which is referred to as the direct association between lifestyle and fatigue. Second, the path between lifestyle and fatigue, passing through inflammation, which is referred to as the indirect association. The summed effect of the direct and indirect paths is referred to as the total path. We used the PROCESS analytic tool developed by Hayes to assess whether inflammation is a mediator in the association between lifestyle and fatigue. This analyses was based on multiple linear regression path analysis [27]. Model 4 of the PROCESS macro version 3.5 for SAS (SAS Institute Inc., Cary, NC, USA) was used to assess indirect, direct, and total effects of the association between lifestyle and fatigue. A 95% percentile bootstrap confidence interval for the indirect effect using 10.000 bootstrap samples was generated.

For the mediation analyses the following sensitivity analyses were done: excluding data of plasma samples that had been stored in the −80 freezer for more than 2 years before analysis, and excluding extreme values (>3SD above the mean). Cytokines were previously shown to remain stable in plasma for a period up to 2 years of storage at −80 °C [43]. Therefore, samples stored >2 years were excluded in a sensitivity analyses.

All data analyses were performed using the statistical software program SAS (version 9.4, SAS Institute Inc.).

## 5. Conclusions

The present study showed that a higher adherence to the WCRF/AICR recommendations at diagnosis was associated with less fatigue among CRC survivors six months after diagnosis, and that inflammation may mediate this association. Future intervention studies should investigate if improving lifestyle directly after diagnosis, for example by improving adherence to WCRF/AICR recommendations, can reduce fatigue during and after treatment.

## Figures and Tables

**Table 1:** Baseline characteristics of colorectal cancer patients stratified for a low, medium, and high World Cancer Research Fund/American Institute for Cancer Research (WCRF/AICR)-score.

Patients Characteristics	Total Population (*n* = 1417)	Low WCRF/AICR-Score ≤3 (Tertile 1) (*n* = 548)	Medium WCRF/AICR-Score >3–3.75 (Tertile 2) (*n* = 485)	High WCRF/AICR-Score ≥3.75 (Tertile 3) (*n* = 384)
Age (years)	66.1 (61.2–71.5)	65.8 (60.4–71.2)	66.5 (61.8–71.5)	66.8 (61.4–72.0)
Sex (female)	514 (36)	175 (32)	170 (35)	169 (44)
Education level *				
low	536 (38)	215 (39)	180 (37)	141 (37)
medium	412 (29)	168 (31)	147 (30)	97 (25)
high	467 (33)	164 (30)	157 (32)	146 (38)
Smoking *				
current	155 (11)	60 (11)	58 (12)	37 (10)
former	811 (58)	322 (60)	280 (58)	209 (55)
never	436 (31)	155 (29)	144 (30)	137 (36)
BMI (kg/m^2^) *	26.4 (24.2–29.4)	28.4 (26.2–31.5)	26.0 (24.1–28.4)	24.3 (22.5–26.4)
Waist circumference (cm)	97 (90–105)	103 (96–110)	95 (89–103)	91 (83–97)
Total moderate-vigorous physical activity (hours/week)	11.0 (5.5–19.0)	8.5 (3.5–17.5)	12 (7.0–20.3)	13 (6.5–21.0)
Dietary intake				
Fruit and vegetable (g/day)	260 (160–366)	187 (121–298)	260 (179–348)	352 (244–447)
Total fibre (g/day)	21 (17–26)	19 (16–24)	21 (17–25)	23 (18–28)
Percentage of total kcal from ultra-processed foods	28 (21–35)	33 (26–39)	27 (21–33)	22 (18–28)
Processed meat (g/day)	29 (13–46)	37 (22–52)	28 (15–46)	14 (4–34)
Red meat (g/day)	38 (23–56)	44 (29–61)	39 (23–56)	31 (17–49)
Sugary drinks (g/day)	72 (14–167)	108 (32–245)	67 (13–152)	42 (0–131)
Alcohol (g/day)	8 (1–21)	12 (2–24)	8 (1–19)	5 (0–17)
Inflammation markers				
IL6 (pg/mL)	1.0 (0.7–1.6)	1.1 (0.7–1.7)	0.9 (0.7–1.6)	0.9 (0.6–1.4)
IL8 (pg/mL)	5.6 (4.2–8.0)	5.6 (4.1–7.9)	5.8 (4.3–8.1)	5.6 (4.3–8.0)
TNFα (pg/mL)	2.0 (1.6–2.6)	2.1 (1.7–2.6)	2.0 (1.5–2.6)	1.9 (1.5–2.4)
hsCRP (μg/mL)	2.7 (1.2–6.5)	3.1 (1.5–7.4)	2.6 (1.2–5.9)	2.2 (1.0–6.3)
Type of Cancer				
colon	929 (66)	357 (65)	322 (67)	250 (65)
rectal	488 (34)	191 (35)	163 (33)	134 (35)
Type of treatment *				
Surgery only	756 (54)	292 (55)	248 (52)	216 (57)
chemotherapy	301 (22)	117 (22)	109 (23)	75 (20)
radiotherapy	224 (16)	89 (17)	72 (15)	63 (16)
chemoradiation	107 (8)	34 (6)	47 (10)	26 (7)
Tumor stage				
I	385 (27)	141 (26)	131 (27)	113 (29)
II	403 (28)	149 (27)	143 (29)	111 (29)
III	629 (44)	258 (47)	211 (44)	160 (42)
Comorbidities (yes) *	968 (69)	389 (71)	337 (70)	242 (63)
Fatigue (yes)	365 (26)	157 (29)	115 (24)	93 (24)
Recurrence within one year (yes)	81 (6)	31 (6)	25 (27)	25 (7)
Daily use of NSAIDs (yes)	110 (8)	45 (8)	37 (8)	28 (7)
Cohort				
COLON	1156 (82)	421 (77)	399 (82)	336 (88)
EnCoRe	261 (18)	127 (23)	86 (18)	48 (13)

Values presented are median (quartile 1–quartile 3) or number (percentages). Abbreviations: IL, interleukin; TNF, tumor necrosis factor; hsCRP, high sensitive c-reactive protein; NSAIDs, non-steroid anti-inflammatory drugs. * = For 2 patients data about education levels was missing, for 15 patients data about smoking status was missing, and for 1 patient BMI was missing, for 29 patients treatment data was missing, for 6 patients data about comorbidities was missing and for 67 patients data about recurrence was missing.

**Table 2:** The association between lifestyle at diagnosis and inflammatory marker levels six months after diagnosis in colorectal cancer patients.

Inflammation 6 Months after Diagnosis		N	Crude Beta 95% CI	Adjusted * Beta 95% CI
IL6				
	WCRF/AICR-score	607	−0.11 (−0.19; −0.04)	−0.10 (−0.17; −0.03)
IL8				
	WCRF/AICR-score	609	−0.05 (−0.10; −0.00)	−0.05 (−0.09; 0.00)
TNFα				
	WCRF/AICR-score	611	−0.08 (−0.11; −0.05)	−0.07 (−0.10; −0.04)
hsCRP				
	WCRF/AICR-score	1116	−0.20 (−0.28; −0.13)	−0.20 (−0.28; −0.12)

* adjusted for age, sex, stage of disease, chemotherapy (yes/no) and cohort. To interpret the beta coefficient of the regression line, the exponential of the beta should be taken (EXP^β^), since a natural log transformation was done on the outcome variable.

**Table 3:** The association between inflammation marker levels and fatigue six months after diagnosis.

Fatigue after 6 Months	N	Crude Beta 95% CI	Adjusted * Beta 95% CI
IL6	607	5.49 (3.03; 7.95)	5.05 (2.54; 7.57)
IL8	609	1.41 (−2.34; 5.15)	1.62 (−2.07; 5.31)
TNFα	611	1.01 (−4.50; 6.51)	3.03 (−2.84; 8.90)
hsCRP	1116	3.57 (2.30; 4.83)	3.57 (2.37; 4.78)

* adjusted for age, sex, stage of disease, chemotherapy (yes/no), and cohort.

**Table 4:** The association between lifestyle and fatigue (total association), divided in an direct path independent of inflammation (direct association), and an indirect path via inflammation (indirect association).

Fatigue 6 Months after Diagnosis		*N*	Β (95%CI)
IL6	Total association	607	−2.17 (−4.44; 0.10)
	Direct association	607	−1.69 (−3.95; 0.56)
	Indirect association	607	−0.48 (−1.05; −0.10)
	Proportion mediated *		22%
IL8	Total association	609	−2.19 (−4.45; 0.08)
	Direct association	609	−2.12 (−4.39; 0.15)
	Indirect association	609	−0.06 (−0.31; 0.12)
	Proportion mediated *		3%
TNFα	Total association	611	−2.19 (−4.45; 0.07)
	Direct association	611	−2.05 (−4.35; 0.24)
	Indirect association	611	−0.14 (−0.69; 0.21)
	Proportion mediated *		6%
hsCRP	Total association	1116	−2.11 (−3.74; −0.48)
	Direct association	1116	−1.42 (−3.05; 0.21)
	Indirect association	1116	−0.68 (−1.10; −0.34)
	Proportion mediated *		32%
Total inflammation (IL6, IL8, TNFα, and hsCRP)	Total association	551	−2.17 (−4.60; 0.25)
	Direct association	551	−1.21 (−3.64; 1.25)
	Indirect association	551	−0.97 (−1.92; −0.21)
	Proportion mediated *		45%

Assessed using the PROCESS method version 3.5 developed by Andrew F. Hayes [27]. Models were adjusted for age, sex, stage of disease, chemotherapy (yes/no), and cohort. In the model for total inflammation all four inflammation markers were entered as potential mediators. * Proportion mediation is calculated by dividing the indirect effect by the total effect, e.g., beta indirect association/beta total association × 100.

## Supplementary Materials

The following are available online at https://www.mdpi.com/2072-6694/12/12/3701/s1, Table S1: Breakdown of the official 2018 WCRF/AICR Score [10] and the percentage of our cohort of colorectal cancer patients that adhered to the specific recommendation, Table S2: Baseline characteristics of the total population, patients of whom hsCRP levels were available and patients of whom IL6 levels were available.
