# Supplementary material for: Inflammation Is a Mediating Factor in the Association between Lifestyle and Fatigue in Colorectal Cancer Patients
Source: Cancers (Basel). 2020 Dec 9;12(12):3701. doi: 10.3390/cancers12123701 (PMC7763620; doi:10.3390/cancers12123701)
Supplement: Supplementary file 1 [file cancers-12-03701-s001.pdf]

cancers

MDPI

Article

Inflammation Is a Mediating Factor in the Association between Lifestyle and Fatigue in Colorectal Cancer Patients

Evertine Wesselink, Harm van Baar, Moniek van Zutphen, Meilissa Tibosch, Ewout A. Kouwenhoven, Eric T. P. Keulen, Dieuwertje E. Kok, Henk K. van Halteren, Stephanie O. Breukink, Johannes H. W. de Wilt, Matty P. Weijenberg, Marlou-Floor Kenkhuis, Michiel G. J. Balvers, Renger F. Witkamp, Fränzel J. B. van Duijnhoven, Ellen Kampman, Sandra Beijer, Martijn J. L. Bours and Renate M. Winkels

Supplementary Table 1 presents the breakdown of the official 2018 WCRF/AICR Score and the percentage of our cohort of colorectal cancer patients that adhered to the specific recommendation.

Table 1. Breakdown of the official 2018 WCRF/AICR Score [10] and the percentage of our cohort of colorectal cancer patients that adhered to the specific recommendation.

2018 WCRF/AICR Recommendations	Operationalisation of Recommendations	Points	Adherence in Total Population (%)
**1.** Be a healthy weight (Lifestyle questionnaire)	BMI (kg/m²)		
	18.5-24.9	0.5	34
	25–29.9	0.25	44
	<18.5 or ≥30	0	22
	Waist circumference (cm)		
	Men: <94; Women: <80	0.5	22
	Men: 94-<102; Women: 80-<88	0.25	28
	Men: ≥102; Women: ≥88	0	50
**2.** Be physically active (SQUASH)	Total moderate-vigorous physical activity (min/week)		
	≥150	1	90
	75-<150	0.5	4
	<75	0	6
**3.** Eat a diet rich in wholegrains, vegetables, fruit and beans (FFQ)	Fruits and vegetables (g/day)		
	≥400	0.5	12
	200-<400	0.25	72
	<200	0	16
	Total fibre (g/day)		
	≥30	0.5	19
	15-<30	0.25	46
	<15	0	35
**4.** Limit consumption of "fast foods" and other processed foods high in fat, starches or sugars (FFQ)	Percent of total kcal from ultra-processed foods (aUPFs)		
	Tertile 1	1	32
	Tertile 2	0.5	33
	Tertile 3	0	36
**5.** Limit consumption of red and processed meat (FFQ)	Total red meat (g/week) and processed meat (g/week)		
	Red meat <500 and processed meat <21	1	8
	Red meat <500 and processed meat 21-<100	0.5	17
	Red meat >500 or processed meat ≥100	0	75

	Total sugar-sweetened drinks (g/day)		
**6.** Limit consumption of sugar-sweetened drinks (FFQ)	0	1	14
	>0-≤250	0.5	70
	>250	0	16
	Total ethanol (g/day)		
**7.** Limit alcohol consumption (FFQ)	0	1	14
	>0-≤28 (2 drinks) males and ≤14 (1 drink) females	0.5	53
	>28 (2 drinks) males and >14 (1 drink) females	0	32
Total score		0–7	

Supplementary Table 2 presents the baseline characteristics of the total population, patients of whom hsCRP levels were available and patients of whom IL6 levels were available and for patient without data about inflammation markers available

Table 2. Baseline characteristics of the total population, patients of whom hsCRP levels were available and patients of whom IL6 levels were available and for patient without data about inflammation markers available.

	**Total Population (*n* = 1417)**	**hsCRP Levels Available (*n* = 1116)**	**IL6 Levels Available (*n* = 607)**	**No hsCRP or IL6 Levels Available (*n* = 866)**
**Age (years)**	66.1 (61.2–71.5)	66.3 (61.4–71.4)	66.8 (61.8–72.5)	65.7 (60.9–71.0)
**Sex (female)**	514 (36)	401 (36)	195 (32)	337 (39)
**Education level***				
**low**	536 (38)	434 (39)	216 (36)	333 (39)
**medium**	412 (29)	318 (29)	192 (32)	245 (28)
**high**	467 (33)	363 (33)	198 (33)	287 (33)
**Smoking***				
**current**	155 (11)	123 (11)	66 (11)	92 (11)
**former**	811 (58)	642 (58)	248 (59)	494 (57)
**never**	436 (31)	336 (31)	179 (30)	279 (32)
**BMI (kg/m²)***	26.4 (24.2-29.4)	26.3 (24.1–29.3)	26.9 (24.5–30.1)	26.1 (24.0–28.7)
**Total moderate-vigorous physical activity (hours/week)**	11.0 (5.5–19.0)	11.0 (5.5–19.0)	12.0 (5.3–21)	11.0 (5.5–18.5)
**Type of Cancer**				
**colon**	929 (66)	743 (67)	394 (65)	564 (65)
**rectal**	488 (34)	373 (33)	213 (35)	302 (35)
**Type of treatment***				
**surgery**	756 (54)	589 (53)	333 (56)	454 (53)
**chemotherapy**	301 (22)	250 (23)	121 (20)	185 (22)
**radiotherapy**	224 (16)	178 (16)	78 (13)	153 (18)
**chemoradiation**	107 (8)	80 (7)	54 (9)	61 (7)
**Tumour stage**				
**I**	385 (27)	296 (27)	177 (29)	225 (26)
**II**	403 (28)	334 (30)	179 (29)	241 (28)
**III**	629 (44)	486 (44)	251 (41)	400 (46)
**Comorbidities (yes)***	968 (69)	767 (69)	455 (75)	559 (65)
**Daily use of NSAIDs (yes)**	110 (8)	90 (8)	42 (7)	70 (8)
**Cohort**				
**COLON**	1156 (82)	931 (84)	388 (64)	784 (91)
**EnCoRe**	261 (18)	184 (17)	219 (36)	82 (9)

Values presented are median (quartile 1 – quartile 3) or number (percentages). Abbreviations: IL, interleukin; TNF, tumour necrosis factor; hsCRP, high sensitive c-reactive protein; NSAIDs, non-steroid anti-inflammatory drugs. For 2 patients data about education levels was missing, for 15 patients data about smoking status was missing and for 1 patients BMI was missing, for 29 patients treatment data was missing and for 6 patients data about comorbidities was missing.

© 2020 by the authors. Licensee MDPI, Basel, Switzerland. This article is an open access article distributed under the terms and conditions of the Creative Commons Attribution (CC BY) license (http://creativecommons.org/licenses/by/4.0/).
